# Paraneoplastic Hypereosinophilia in Locally Advanced Clear Cell Renal Carcinoma

**DOI:** 10.7759/cureus.39726

**Published:** 2023-05-30

**Authors:** Halil İbrahim Ellez, Esra Bayram, Erkut Demirciler, Hüseyin Salih Semiz

**Affiliations:** 1 Medical Oncology, Dokuz Eylul University, İzmir, TUR; 2 Internal Medicine, Dokuz Eylul University, İzmir, TUR; 3 Institute of Oncology, Dokuz Eylül University, İzmir, TUR

**Keywords:** cytokin, locally advanced tumors, renal cell carcinoma (rcc), hyper-eosinophilia, paraneoplastic syndromes

## Abstract

Renal cell carcinoma (RCC) can cause various paraneoplastic syndromes, including metabolic and hematologic disturbances. Paraneoplastic hypereosinophilia has been reported in a variety of hematologic and solid tumors. Hypereosinophilia due to RCC is very rare and is only available as case reports in the literature. A 66-year-old male patient's thoracoabdominal computed tomography (CT) performed showed an increase in size in the right kidney and a heterogeneous contrasting solid mass of approximately 12 cm × 9 cm, which formed lobulations in its contours. The patient was diagnosed with clear-cell renal carcinoma as a result of a kidney biopsy. In the patient with stage cT4NxM0, the leukocyte count was 40.000/µl and the eosinophil count was 20% in biochemical tests. With these results, the patient was evaluated as having severe paraneoplastic hypereosinophilia due to RCC. The patient was started on sunitinib 50 mg for two weeks on/one week off. No symptoms were observed due to hypereosinophilia. In the evaluation made two weeks after the start of the treatment, it was observed that eosinophil levels decreased to normal rates. Paraneoplastic hypereosinophilia due to renal cell carcinoma may indicate poor prognosis and rapid disease progression. Myelosuppressive therapy is required for symptomatic patients.

## Introduction

Paraneoplastic syndromes occur with the release of peptides, hormones, or cytokines secreted from tumor tissue or due to cross-reactivity between tumor cells and normal tissues [1]. Paraneoplastic syndromes are seen in approximately 40% of renal cell carcinomas (RCC) [2]. The most common paraneoplastic syndrome in RCC is fatigue. Other findings related to hypercalcemia, hypertension, and ectopic hormone secretion are also frequently observed. The most common hematological disorder in RCC is polycythemia due to an increase in hypoxia-inducible factor (HIF). Hypereosinophilia due to RCC is very rare and is only available as case reports in the literature. We will also describe a case describing asymptomatic hypereosinophilia in locally advanced renal cell carcinoma and its condition after sunitinib use.

## Case presentation

**Figure 1:**
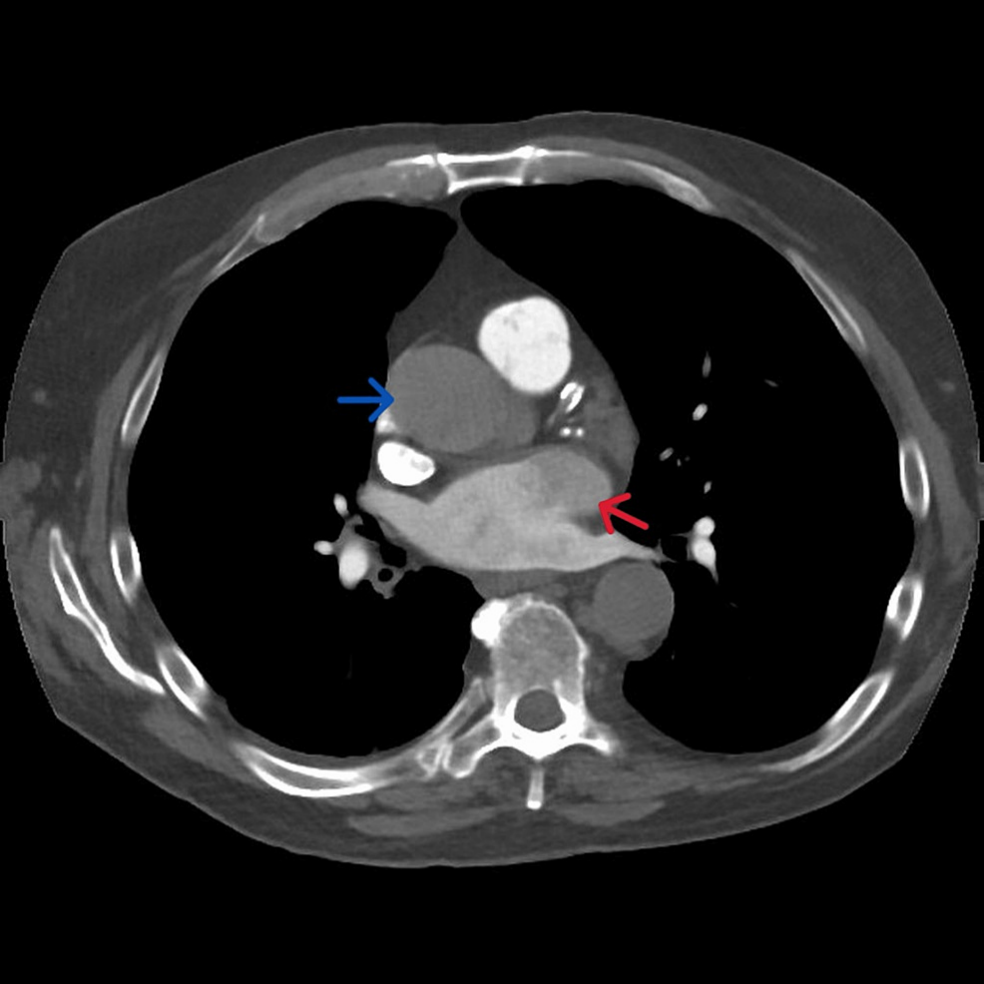
Thrombus in the right atrium and vena cava seen on CT (red arrow: thrombus in vena cava; blue arrow: thrombus in right atrium)

**Figure 2:**
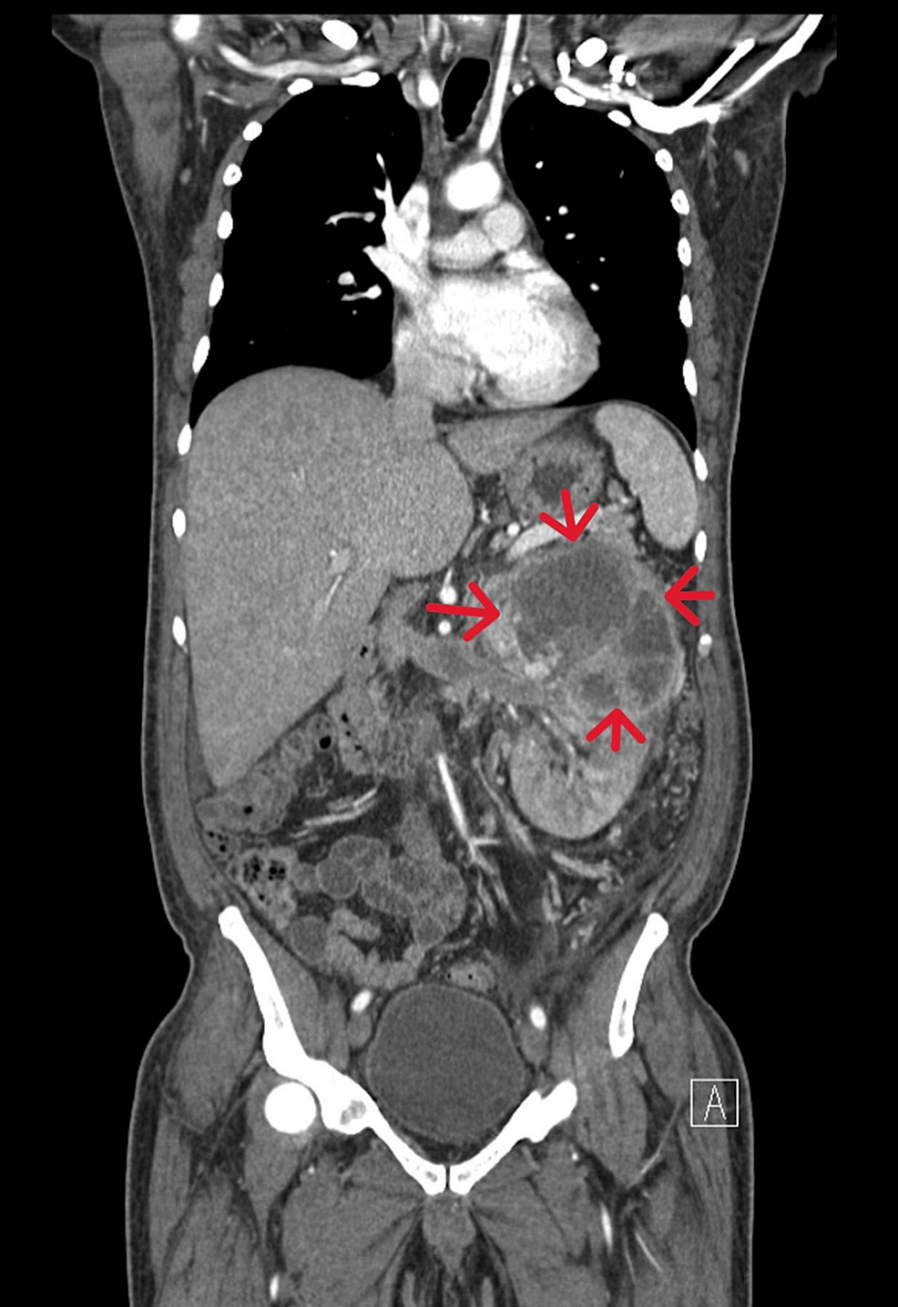
Mass in the right kidney viewed in the coronal plane on computed tomography (red arrows: renal mass in right kidney)

A 66-year-old male patient, on thoracoabdominal computed tomography (CT) performed for edema in both legs, weakness, and weight loss, which started in May 2022, showed an increase in size in the right kidney and a heterogeneous contrasting solid mass of approximately 12 cm × 9 cm, which formed lobulations in its contours (Figure 1). On the right, a tumor thrombus is seen in the renal vein and the inferior vena cava. The tumor thrombus reaches up to the atrium in the right part of the heart (Figure 2).

**Table 1:** Case laboratory parameters at the diagnosis WBC: white blood count, NEU: neutrophil, LYM: lymphocyte, EOS: eosinophil, MONO: monocyte, HGB: hemoglobin, PLT: thrombocyte

Laboratuar parameters	Value (/µl)	Normal range (/µl)
WBC	40,000	4000–11,000
NEU	20,000	1500–8000
LYM	7000	1000–4800
EOS	8000	0–500
MONO	5000	0–1000
HGB	8.2	11.2–15.4
PLT	558,000	150,000–400,000

**Table 2:** Case laboratory parameters at the first month treatment WBC: white blood count, NEU: neutrophil, LYM: lymphocyte, EOS: eosinophil, MONO: monocyte, HGB: hemoglobin, PLT: thrombocyte

Laboratuar parameters	Value (/µl)	Normal range (/µl)
WBC	10,300	4000–11,000
NEU	7000	1500–8000
LYM	2000	1000–4800
EOS	300	0–500
MONO	1000	0–1000
HGB	9.4	11.2–15.4
PLT	350,000	150,000–400,000

The patient was diagnosed with clear-cell renal carcinoma as a result of a kidney biopsy performed in June 2022. An intermediate stage was observed according to the Memorial Sloan-Kettering Cancer Center's (MSKCC) risk classification. In the patient with stage cT4NxM0, the leukocyte count was 40,000/µl and the eosinophil count was 20% in biochemical tests. He also had anemia and thrombocytopenia. Bone marrow aspiration and biopsy were performed on the patient with the pre-diagnosis of myeloproliferative disease and paraneoplastic syndrome associated with RCC concurrently with the diagnosis of RCC. BCR/ABL (P210), Janus Kinase 2 (JAK2) V617F mutation, and calreticulin (CALR) mutation were studied from peripheral blood and bone marrow aspiration, and the results were negative. Apart from this, anti-nuclear antibody (ANA) (immunofixation), anti-neutrophil cytoplasmic antibody (ANCA) (immunofixation), and C3 and C4 levels for rheumatologic paraneoplastic syndromes were not significant. It was evaluated by urology as unsuitable for nephrectomy.

**Figure 3:**
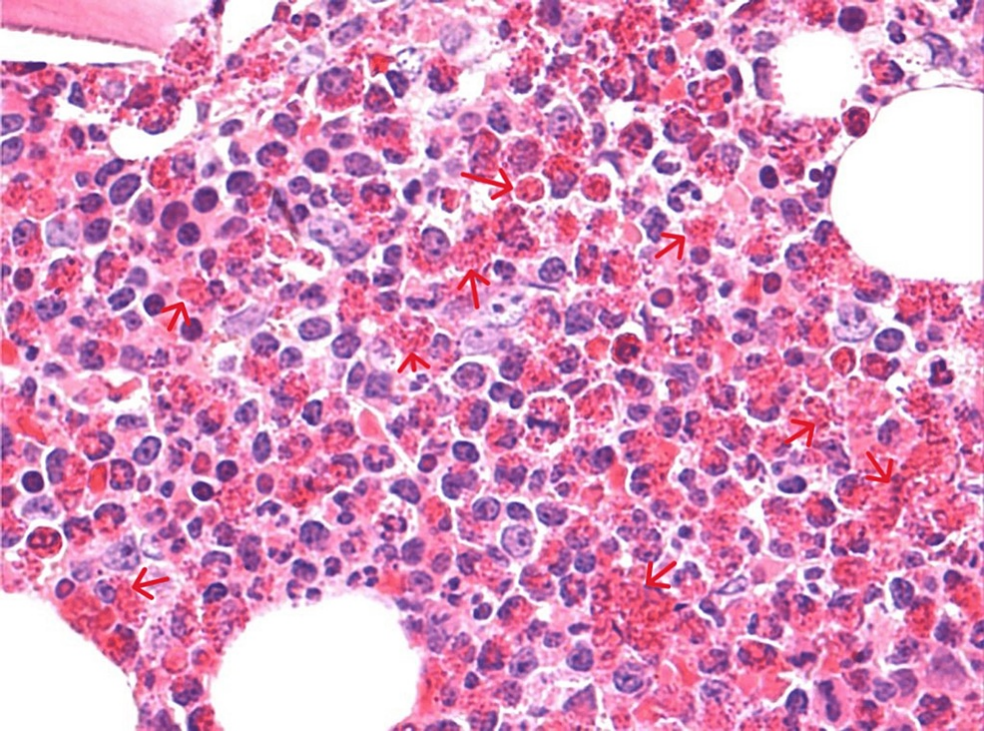
Hypereosinophilia in bone marrow aspiration (BM biopsy, hematoxylin and eosin, original magnification ×400) Red arrow: eosinophil in bone marrow aspiration

The bone marrow biopsy result was consistent with hypercellular bone marrow. Dysplastic cells were not observed, and the ratio of the myeloid/erythroid series was increased in favor of the myeloid series (M:E approximately 7-8:1). Eosinophil leukocytes at different stages of maturation were slightly increased in focal areas (Figure 3). The serum tryptase value was 3 ng/ml (normal range: 0-5 ng/ml). No positive results were found in stool or blood tests for allergic infections. FIP1L1/PDGFR receptor mutations were not detected. CD3 and CD4 levels could not be measured because they were not studied in our country.

The patient's thrombus, starting from the inferior vena cava and extending to the right atrium, was not associated with hypereosinophilia. Due to problems in the social security system in Turkey, the patient was started on sunitinib 50 mg for two weeks on/one week off. No symptoms were observed due to hypereosinophilia. In the evaluation made two weeks after the start of the treatment, the leukocyte count was 20,000 µ/l and the eosinophil level was 10%. In the first-month evaluation made in July 2022, it was observed that eosinophil levels decreased to normal rates. With these results and treatment response, the patient was evaluated as having severe paraneoplastic hypereosinophilia due to RCC.

## Discussion

Hypereosinophilia is most commonly seen in allergic diseases or parasitic infections. In addition, hypereosinophilia can be seen in drugs, some pulmonary and gastrointestinal diseases, and malignancies [3]. Mild to moderate hypereosinophilia (500-1500 µ/l) is seen in approximately 5% of all malignancies. In recent years, some molecular disorders that cause hypereosinophilia have been detected by molecular tests. The most common and best-revealing of these are those seen as a result of platelet-derived growth factor receptor (PDGFR) rearrangement [4]. Severe hypereosinophilia due to malignancy (5000 µ/l≤) is frequently seen in bronchial carcinomas, gastrointestinal cancers, prostate cancer, and sarcomas [5-7]. Hypereosinophilia due to malignancy is thought to develop due to cytokines such as interleukin-3 (IL-3), interleukin-5 (IL-5), granulocyte-stimulating factor (G-CSF), and granulocyte-macrophage stimulating factor (GM-CSF) [8]. In this case, we cannot comment on this issue since the cytokine level could not be studied under the conditions in Turkey. In a case report made in Germany in 2012, hypereosinophilia was detected in a patient with metastatic RCC, and G-CSF and GM-CSF levels in the peripheral blood were found to be high and decreased to an undetectable level in the blood with treatment [9]. The cytokine levels measured in hypereosinophilia due to malignancy may not always be significant in the diagnosis and treatment follow-up since it is impossible to determine the source of these cytokines due to the cytokines secreted by the increased eosinophils [10].

It has been shown that hypereosinophilia is observed due to tumor necrosis, and, accordingly, the lymphocyte-tumor relationship clusters around tumor necrosis [11]. In our case, tumor necrosis could not be differentiated by imaging methods. No tumor necrosis was observed in the kidney biopsy. Since the patient did not undergo a nephrectomy, we cannot comment on this. Paraneoplastic eosinophilia is usually mild without any clinical symptoms. However, thromboembolic events and related organ damage can be seen when the eosinophil count is 25,000 µ/l and above. In our case, the eosinophil level was 8000 µ/l at the time of diagnosis. The tumor thrombus had no relationship with eosinophilia. In our case, a significant increase in neutrophils and other series could not be proven because a nephrectomy could not be performed on the patient. However, hypereosinophilia is thought to contribute to this condition. Paraneoplastic eosinophilia is an indicator of a poor prognosis and, generally, of metastatic disease [12]. In our case, the patient is in a locally advanced stage and has been followed up for two months under treatment. A clinical response was observed with treatment. However, the patient cannot reach standard primary care treatment due to treatment payment problems within the scope of social security in our country. Because of this situation, the prognosis is estimated to be poor. Symptomatic paraneoplastic eosinophilia can be treated with drugs that cause decreased production and function of eosinophilic granulocytes, including glucocorticoids, hydroxyurea, and vincristine [13]. Apart from this, with treatment for the disease (surgery, chemotherapy, and radiotherapy), there may be a regression in both the number of eosinophils and the symptoms. In our case, a decrease of approximately 50% was detected in the eosinophil value measured in the second week after the treatment, and it returned to normal levels in the first-month control. During the follow-up, it was observed that the weight loss stopped and the constitutional symptoms decreased significantly. This suggests that eosinophil levels may be associated with treatment response in patients with paraneoplastic eosinophilia.

## Conclusions

In the literature review on paraneoplastic hypereosinophilia in RCC, we were able to detect eight cases in a total of five studies. All patients seen in these studies were in the metastatic stage. In our study, our patient was in a locally advanced stage and received a dramatic response in the first month of treatment. Paraneoplastic hypereosinophilia due to renal cell carcinoma may indicate poor prognosis and rapid disease progression. Myelosuppressive therapy is required for symptomatic patients.
